# Supplementary material for: Dendritic spikes in hippocampal granule cells are necessary for long-term potentiation at the perforant path synapse
Source: eLife. 2018 Mar 26;7:e35269. doi: 10.7554/eLife.35269 (PMC5896953; doi:10.7554/eLife.35269)
Supplement: Transparent reporting form [file elife-35269-transrepform.docx]

***eLife’s* transparent reporting form**

We encourage authors to provide detailed information *within their submission* to facilitate the interpretation and replication of experiments. Authors can upload supporting documentation to indicate the use of appropriate reporting guidelines for health-related research (see [EQUATOR Network](http://www.equator-network.org/%20)), life science research (see the [BioSharing Information Resource](https://biosharing.org/)), or the [ARRIVE guidelines](http://www.plosbiology.org/article/info:doi/10.1371/journal.pbio.1000412) for reporting work involving animal research. Where applicable, authors should refer to any relevant reporting standards documents in this form.

If you have any questions, please consult our Journal Policies and/or contact us: [editorial@elifesciences.org](mailto:editorial@elifesciences.org).

**Sample-size estimation**

- You should state whether an appropriate sample size was computed when the study was being designed
- You should state the statistical method of sample size computation and any required assumptions
- If no explicit power analysis was used, you should describe how you decided what sample (replicate) size (number) to use

Please outline where this information can be found within the submission (e.g., sections or figure legends), or explain why this information doesn’t apply to your submission:

We used G*power program (Faul et al., 2009; http://www.gpower.hhu.de/) for estimating sample size.

To assess a significant difference in mean value between two independent groups with a power of 0.08, sample-size of 5 cells in each group is needed when Mann-Whitney U test is used with parameter values that are typical for the present study (α = 0.05, the mean of test group = 0.2 x mean of control group, the standard deviation (SD) < 0.4 x mean).

Accordingly, we obtained data from more than 5 cells when statistical significance was tested. Sample size were indicated in the text and figure legends.

**Replicates**

- You should report how often each experiment was performed
- You should include a definition of biological versus technical replication
- The data obtained should be provided and sufficient information should be provided to indicate the number of independent biological and/or technical replicates
- If you encountered any outliers, you should describe how these were handled
- Criteria for exclusion/inclusion of data should be clearly stated
- High-throughput sequence data should be uploaded before submission, with a private link for reviewers provided (these are available from both GEO and ArrayExpress)

Please outline where this information can be found within the submission (e.g., sections or figure legends), or explain why this information doesn’t apply to your submission:

We reported number of cells for each experiment in figure legends and the text.

We performed experiments 4–5 days a week and usually obtained 2 data per one day. However, it varies a lot for each experiment depending on circumstances that are not related with science, such as problems in animal supply. Since such variation does not have any implication about the data interpretation, we did not state how often each experiment was performed.

For replicability, experimental procedures were described in detail in Materials and Methods.

We excluded data for analysis when the resting membrane potential was more positive than −70 mV. Experiments were stopped if the resting membrane potential or input resistance changed by more than 20% during the recording.

We stated these criteria in Materials and Methods.

**Statistical reporting**

- Statistical analysis methods should be described and justified
- Raw data should be presented in figures whenever informative to do so (typically when N per group is less than 10)
- For each experiment, you should identify the statistical tests used, exact values of N, definitions of center, methods of multiple test correction, and dispersion and precision measures (e.g., mean, median, SD, SEM, confidence intervals; and, for the major substantive results, a measure of effect size (e.g., Pearson's r, Cohen's d)
- Report exact p-values wherever possible alongside the summary statistics and 95% confidence intervals. These should be reported for all key questions and not only when the p-value is less than 0.05.

Please outline where this information can be found within the submission (e.g., sections or figure legends), or explain why this information doesn’t apply to your submission:

For comparisons of paired data, a two-sided non-parametric Wilcoxon signed-rank test was performed. For comparisons of unpaired data, a two-sided non-parametric Wilcoxon rank sum test was used. For comparisons of more than two groups, a Kruskal-Wallis test with Dunn’s multiple comparisons for post-hoc testing was used.

Raw data were presented in figures whenever informative to do so, especially when the data in the control and experimental condition were obtained from the same cell. Mean ± SEM were presented in bar graphs and the text. In addition, we presented individual data points on top of bar graphs for each group. We identified the statistical test methods, number of cells tested, and p-values for each experiment.

We stated above information in the data analysis section.

p-values for all key questions were reported in the text and figures.

(For large datasets, or papers with a very large number of statistical tests, you may upload a single table file with tests, Ns, etc., with reference to sections in the manuscript.)

**Group allocation**

- Indicate how samples were allocated into experimental groups (in the case of clinical studies, please specify allocation to treatment method); if randomization was used, please also state if restricted randomization was applied
- Indicate if masking was used during group allocation, data collection and/or data analysis

Please outline where this information can be found within the submission (e.g., sections or figure legends), or explain why this information doesn’t apply to your submission:

n/a

**Additional data files (“source data”)**

- We encourage you to upload relevant additional data files, such as numerical data that are represented as a graph in a figure, or as a summary table
- Where provided, these should be in the most useful format, and they can be uploaded as “Source data” files linked to a main figure or table
- Include model definition files including the full list of parameters used
- Include code used for data analysis (e.g., R, MatLab)
- Avoid stating that data files are “available upon request”

Please indicate the figures or tables for which source data files have been provided:

Source data has been provided for data in all figures.
